# Facial electromyography during exercise using soft electrode array: A feasibility study

**DOI:** 10.1371/journal.pone.0298304

**Published:** 2024-02-15

**Authors:** Rawan Ibrahim, Itay Ketko, Mickey Scheinowitz, Yael Hanein

**Affiliations:** 1 Department of Biomedical Engineering, Tel Aviv University, Tel Aviv, Israel; 2 Medical Corps, Israel Defense Forces, Ramat Gan, Israel; 3 Sylvan Adams Sports Institute, School of Public Health, Sackler Faculty of Medicine, Tel Aviv University, Tel Aviv, Israel; 4 School of Electrical Engineering, Tel Aviv University, Tel Aviv, Israel; 5 Sagol School of Neuroscience, Tel Aviv University, Tel Aviv, Israel; 6 X-trodes, Herzelia, Israel; 7 Tel Aviv University Center for Nanoscience and Nanotechnology, Tel Aviv University, Tel Aviv, Israel; Universita Politecnica delle Marche Facolta di Ingegneria, ITALY

## Abstract

The use of wearable sensors for real-time monitoring of exercise-related measures has been extensively studied in recent years (e.g., performance enhancement, optimizing athlete’s training, and preventing injuries). Surface electromyography (sEMG), which measures muscle activity, is a widely researched technology in exercise monitoring. However, due to their cumbersome nature, traditional sEMG electrodes are limited. In particular, facial EMG (fEMG) studies in physical training have been limited, with some scarce evidence suggesting that fEMG may be used to monitor exercise-related measurements. Altogether, sEMG recordings from facial muscles in the context of exercise have been examined relatively inadequately. In this feasibility study, we assessed the ability of a new wearable sEMG technology to measure facial muscle activity during exercise. Six young, healthy, and recreationally active participants (5 females), performed an incremental cycling exercise test until exhaustion, while facial sEMG and vastus lateralis (VL) EMG were measured. Facial sEMG signals from both natural expressions and voluntary smiles were successfully recorded. Stable recordings and high-resolution facial muscle activity mapping were achieved during different exercise intensities until exhaustion. Strong correlations were found between VL and multiple facial muscles’ activity during voluntary smiles during exercise, with statistically significant coefficients ranging from 0.80 to 0.95 (p<0.05). This study demonstrates the feasibility of monitoring facial muscle activity during exercise, with potential implications for sports medicine and exercise physiology, particularly in monitoring exercise intensity and fatigue.

## Introduction

The use of non-invasive wearable sensors for real-time monitoring during physical exercise has been studied extensively [1]. Real-time and continuous monitoring of physiological variables during exercise provides valuable information with implications for optimizing athlete’s training, enhancing performance, avoiding over-training, preventing injuries, and post-injury rehabilitation. One of the most researched applications of wearable sensors is the identification of athletes’ exercise intensity and fatigue evaluation during exercise [2–4]. The link between exercise intensity, exercise-related fatigue, and facial sEMG was studied over the last two decades, pointing to strong correlations between facial muscles and fatigue-related measures [5–11]. For example, it has been suggested that epicranius (frowning expression) [6, 10], zygomatic major and masseter muscle (jaw clenching) activation [10] are positively correlated with the degree of exertion and fatigue-related measures during exercise and physically demanding tasks [8, 10]. In a recent review, focusing on applications of facial sEMG recording, it was summarized that mapping emotional expression has a vast potential in a quantitative evaluation of fatigue, resulting from physiological labor and/or psychological stress [12].

Video analysis of facial expressions combined with machine learning algorithms is the most commonly used method for studying the expression of fatigue in the face [10, 13]. Several recent studies showed the use of video analysis during exercise (e.g. [14, 15]). However, to achieve high-resolution visual access, facial video analysis during exercise requires the athlete to set their head in a non-convenient fixed position in front of a camera without the ability to move freely when cycling, running, or performing resistance exercises. Additionally, as facial expressions depend on a complex activation of multiple facial muscles [16], a fundamental limitation of video or image analysis is to identify which muscles have been activated.

Considering the above challenges, continuous and direct measurement of muscle-related fatigue during exercise by conventional facial surface electromyography (sEMG) recordings was considered [17–23]. However, conventional sEMG monitoring during exercise suffers from major technological challenges. An optimal electrode-skin electric coupling commonly mandates conductive electrolytic gel between the skin and the metal electrode [24]. Such wet electrodes, which are widely used in clinical work, tend to dry and usually allow only short-term signal recording, typically at low resolution, as the electrode’s surface covers a large skin area. sEMG recordings are particularly prone to instability and mechanical artifacts during dynamic exercise due to the cumbersome wet electrodes, which are typically connected via wires to relatively static and large recording equipment. Thus, accurate sEMG signal recording is still limited to a clinic or a laboratory setting and requires experienced technicians. Clearly, for high-resolution sEMG during exercise, an sEMG system should be portable, soft, easy to apply, and with minimal use of wires to avoid interference with the subject’s training, while also presenting stability against sweat and movements [1, 25, 26].

Wearable sEMG with high resolution and stability against sweating and movements suitable for convenient application to the face opens a wide range of opportunities, particularly in examining subjects’ facial expressions during intense exercise. In this investigation, we used a novel soft sensor for the high-resolution recording of surface electromyography from the face [27, 28] to resolve the challenges described above. The technique utilizes a soft, high-density printed dry electrode array. Previously, these printed electrode arrays were demonstrated to enable continuous and accurate measurement of sEMG signals in a convenient and simple-to-use manner under resting conditions [29]. Here, for the first time, this new soft sEMG sensor was tested during exercise. The aim of the current study was to assess the feasibility of recording facial muscle activity during exercise and to provide a proof of concept that facial sEMG can be used to quantify effort during exercise.

## Materials and methods

The study was carried out at the Sylvan Adams Sports Institute at Tel Aviv University, Israel. The study protocol was approved by the Institutional Ethics Committee of Tel Aviv University (approval #0000447-4) and all participants provided written informed consent prior to participation. Participant recruitment for this study started on February 1, 2022, and concluded on April 12, 2022. Any identifiable participant in this study has given written consent for publication.

### Multi-array sEMG system

**Fig 1:**
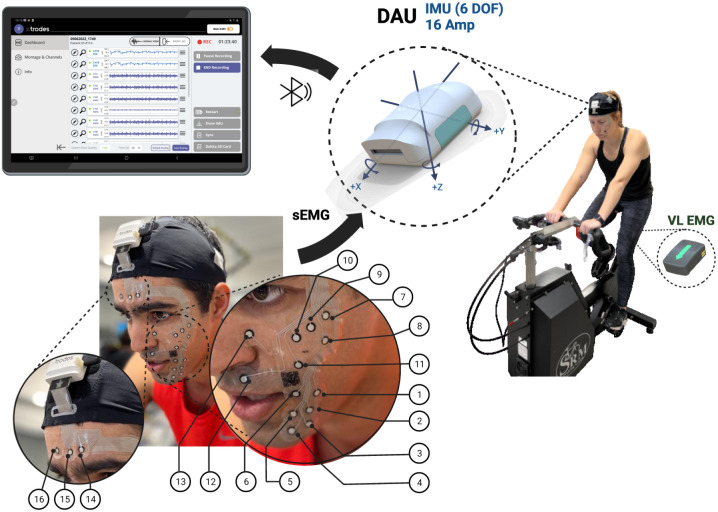
Experimental setup. The sEMG multi-electrode array sticker placement on a participant, with the channel numbers corresponding to each electrode, in both upper and lower facial regions. Also shown are the VL EMG unit and the DAU supporting 16-channel facial EMG recording and acceleration data collection of the head.

The electrode arrays and the miniature data acquisition unit (DAU) that were tested in this study were purchased from X-trodes Inc. The sensors were previously described in [27, 28]. The sensors were developed for various applications, such as sleep monitoring, emotional affect detection, and smile mapping [29–31]. Briefly, the sensor utilizes a high-density skin electrode array (16 electrodes), made of conductive carbon and silver ink, embedded in soft support (Fig 1). The softness of the arrays allows good mechanical coupling to the subject’s skin, which does not disrupt or restrict the subject’s movement. The electrodes are dry (no coupling medium is required) and allow long-term accurate recording. This innovative manufacturing method allows flexibility in the location, size, and density of the electrode array, so that the electrode array may be shaped per the required application. To enable convenient recording of sEMG in natural settings during everyday life activities, the electrode array is connected to a wireless data acquisition unit (DAU, X-trodes Inc.) The DAU is a wireless system containing a lightweight amplifier and transducer that supports up to 16 unipolar channels (2 μV noise RMS, 0.5-700 Hz) with a sampling rate of 4000 S/s, 16-bit resolution and input range of ± 12.5 mV. A 620 mAh battery supports DAU operation for up to 16 hours. The amplified analog signal is converted into a digital signal and transmitted via Bluetooth (BT) protocol to a recording unit. The DAU is controlled by an Android application in which the signal can be viewed continuously. The data are stored on a built-in SD card and can be uploaded to a designated Cloud-based platform for further analysis. The DAU also includes a 3-axis inertial sensor to measure the acceleration of the head with a sampling rate of 1000 S/s.

### Experimental outline

The Facial sEMG array was positioned on the participant’s left side of the face (see Fig 1), covering the forehead, zygomaticus, and lower face regions. The monitored locations are described in Table 1. The same electrode array design was used for all the experiments without accounting for anatomical facial differences. A commercial ground plate electrode (Natus) with a contact area of 40 X 50 mm was placed at the cervical spine. The sensor was connected to the DAU and was attached to a custom-made headband. Prior to each test, a calibration phase was performed in which participants were asked to mimic several facial expressions that were expected to be seen during exercise (i.e. a wide smile, frowning, closing the eyes, an expression of great pain or exertion).

**Table 1:** Facial EMG channels and corresponding facial regions (lower face—LF, upper face—UF) and monitored location.

Electrode channel	Location	Facial region
Ch1-6	Depressor muscles	LF
Ch8	Zygomaticus major	LF
Ch7, 9-10	Zygomaticus minor	LF
Ch11	Levator anguli oris	LF
Ch12	Orbicularis oris	Perioral
Ch13	Nasalis	Nose
Ch14	Corrugator supercilii	UF
Ch15	Procerus	UF
Ch16	Frontalis	UF

In addition, sEMG of the Vastus Lateralis (VL) was measured continuously during the exercise, in parallel to the facial sEMG, by a single EMG unit (Tringo, Delsys) with a sampling rate of 1500 S/s. The electrodes were attached to the VL muscle according to SENIAM standard (http://seniam.org/sensor_location.htm).

### Exercise stress test

Six healthy young and recreationally active volunteers (age 25.7 ± 5.7 y, BMI 21.4 ± 3.1 kg/m^2^; 5 females) participated in the study. Participants performed an incremental cycling test to exhaustion on a cycle ergometer (Schoberer Rad-Messtechnik, SRM GmbH, Germany). Starting from 3 min of rest, and followed by a 3 min warm-up stage, the participants were instructed to cycle at a constant pedal frequency of 70 revolutions per minute (RPM), starting with 50 W, while ramping 25 W at each stage, lasting 3 min each. Above the point of onset of blood lactate accumulation (that was defined as 4 mmol by measuring the lactate levels with blood drawn from the participant’s fingertip at the end of each stage), the 25 W resistance increments lasted 1 min, until exhaustion. Exhaustion was defined as a pedal frequency of fewer than 60 RPM for more than 5 s, despite the participant’s effort to continue pedaling. Following cessation, the participants were instructed to stay seated for 3 min for a recovery period, while sEMG was continuously monitored. Throughout the exercise, the participants were instructed to remain silent to avoid unnecessary activation of facial muscles. To test the sensor’s feasibility of recording facial sEMG during different exercise intensities, while amplifying the signal, the participants were instructed to smile at the end of each stage of the test.

### Data analysis

Raw facial EMG data were filtered using a 4th order Butterworth bandpass filter (BPF), with a frequencies band of 30-350 Hz with a hamming window, to attenuate non-sEMG components. A notch filter with 50 Hz cut-off frequency and q-factor of 100 was used to remove power-line interference. Root mean square (RMS) values of the last 30 s of each stage were calculated and normalized to the individual’s maximal RMS value (obtained at the last stage). Raw EMG data of the VL muscle were filtered similarly, apart from the fact that the BPF was set to the frequency band of 20-400 Hz. Voluntarily smile events were segmented by using manual annotations, labeled during each stage of the exercise test. Annotations were corrected during the post-processing phase as follows: a moving standard deviation (STD) window of 4 s around the manual timestamp, with a 0.2 s overlap, was applied for all 16 channels. For each event, the beginning of the smile was defined as the first time that the STD reached a threshold *T*_0_, and the smile’s end was defined as the first time the STD of all channels was below the threshold. The RMS value of each smile segment along the exercise was calculated for each channel, and was normalized to the RMS value of the smile evoked at the resting stage, in order to overcome the variability of the individuals’ smile pattern. Neutral expressions segments were chosen as the segments with minimal noise before a smiling event (about 30 seconds before the smile event). The RMS of each neutral expression segment was calculated and normalized similarly for the smile segments.

Since each participant performed a different number of exercise stages until exhaustion, averaging the participants’ RMS values, for both facial and VL data, was conducted according to exercise intensities. The exercise duration was divided into five bins, with each bin representing an exercise intensity range calculated as a percentage of maximal power that was reached towards the end of the test: 0-20%, 20-40%, 40-60%, 60-80%, and 80-100%. For each participant, intensities were normalized using min-max normalization of power output. Segments’ RMS values were aggregated into the relevant bin and then averaged. To explore facial expression variations at different exercise intensities until exhaustion, the neutral facial expression ratio (NFER) was calculated for each exercise intensity bin by dividing the RMS within bin (i) by the previous RMS at bin (i-1). This approach was chosen to evaluate the signal quality in addition to the signal-to-noise (SNR) analysis. NFER considers the signal’s stability while increasing the exercise intensity, which brings about higher mechanical artifacts. In addition, to test the feasibility of mapping specific facial muscles expressed during exercise, an adapted fastICA algorithm, as was used in previous studies [29], was applied to the filtered data. MATLAB software (Mathworks Inc. Natick, MA, USA) was used for data analysis and plotting, Fig 1 was created with BioRender.com.

## Results

The soft facial sEMG array was tested during an incremental cycling exercise test until exhaustion on six participants. The test lasted 23.5±4.8 min, during which time the participants reached a maximal power output of 191±34 W with a corresponding maximal heart rate of 179±14 beats per minute (BPM). The experimental setup is depicted in Fig 1.

**Fig 2:**
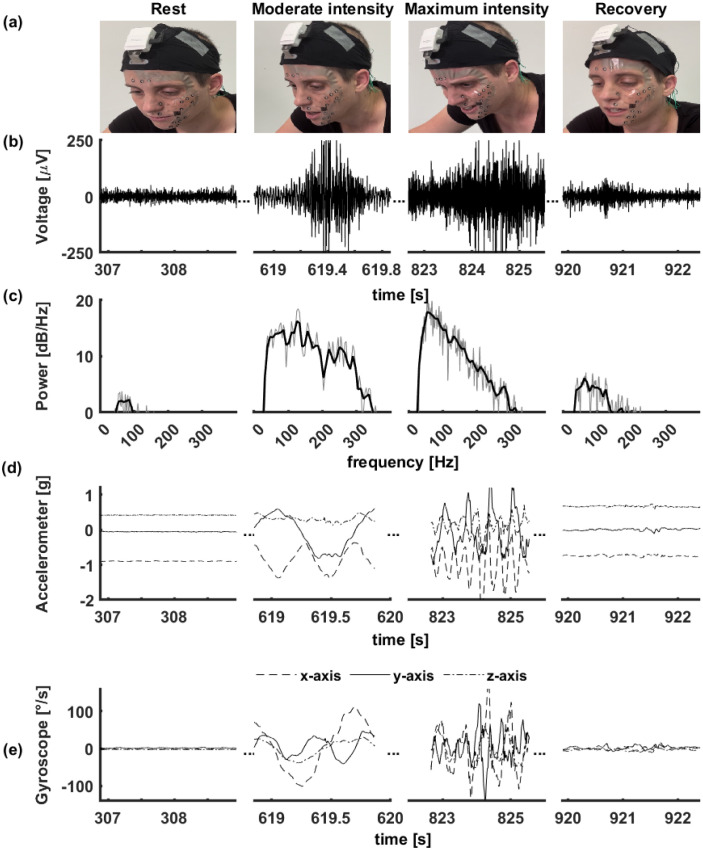
Facial sEMG during different exercise intensities. Capture of facial expression **(a)**; Raw signal from the zygomaticus major muscle (Ch8) **(b)**; The estimated Wlech’s power spectral density (PSD) **(c)**; Corresponding accelerometer and gyroscope 3-axis data **(d and e)**.

Facial EMG signals showed a quick stabilization for all 16 channels shortly after the sensor’s initial set-up. The baseline-noise root mean square (RMS), calculated during neutral expression at rest prior to the test, was 16.00±4.50 *μ*V. The noise RMS peaked during the maximal exercise intensity to a value of 36.44±13.94 *μ*V and decreased after 3 min of recovery to 18.17±4.42 *μ*V. All 16 channels remained stable throughout the test and detachments of the electrode arrays were not observed. Such stability was achieved, even when significant sweating was apparent, and when the participants breathed heavily and made significant facial expressions (typically towards the end of the test due to extensive effort). All participants reported that the sensor was comfortable, and its use did not interfere with cycling, even at high intensities. Fig 2 demonstrates the ability of the sensor to record an sEMG signal of neutral facial expressions that varied throughout the exercise as its intensity was increased (Fig 2a). For example, the raw signal obtained from the zygomaticus major muscle (channel #8) at different intensities, in correspondence to the depicted facial expression (Fig 2a), is shown in Fig 2b, together with the signal’s estimated Welchs power spectral density (Fig 2c). The clean sEMG data recorded with the soft sensors was obtained despite significant head movements, as evident by the linear and angular acceleration data derived from the DAU’s embedded inertia sensors (Fig 2d and 2e).

Table 2 summarizes the neutral facial expression ratio (NFER) values which were calculated for all 16 channels throughout the test. NFER values were distributed in the range of 0.93 to 1.33. In general, NFER values were close to 1 with a relatively small standard deviation, pointing to a stable and robust signal in the presence of disturbance during the exercise. Towards the end of the test (maximal power output), the signal tends to be more susceptible to interference or distortion due to the increase in exercise intensity.

**Table 2:** Neutral facial expression ratio (NFER) calculated for all 16 channels during the test, indicating signal’s stability throughout the intensity range (N = 6).

	$\frac{{\text{RMS}}_{\text{bin}(\text{I})}}{{\text{RMS}}_{\text{bin}(\text{I}-1)}}$						
	$\frac{\text{Warmup}}{\text{Rest}}$	$\frac{0-20\%}{\text{Warmup}}$	$\frac{20-40\%}{0-20\%}$	$\frac{40-60\%}{20-40\%}$	$\frac{60-80\%}{40-60\%}$	$\frac{80-100\%}{60-80\%}$	$\frac{\text{Recovery}}{80-100\%}$
**Ch1**	1.68±0.31	0.97±0.09	1.04±0.19	1.16±0.13	1.05±0.16	1.18±0.23	0.49±0.14
**Ch2**	1.66±0.33	1.01±0.06	0.99±0.10	1.13±0.11	1.07±0.25	1.27±0.34	0.49±0.14
**Ch3**	1.68±0.33	1.00±0.08	1.02±0.11	1.26±0.10	1.06±0.20	1.23±0.35	0.48±0.14
**Ch4**	1.66±0.30	0.98±0.07	1.01±0.09	1.18±0.17	1.11±0.35	1.33±0.48	0.46±0.14
**Ch5**	1.68±0.30	0.93±0.14	1.01±0.12	1.14±0.11	1.12±0.24	1.21±0.27	0.51±0.12
**Ch6**	1.65±0.32	1.02±0.05	1.07±0.28	1.13±0.11	1.02±0.15	1.21±0.24	0.54±0.15
**Ch7**	1.78±0.37	0.98±0.10	0.99±0.13	1.12±0.11	1.03±0.16	1.13±0.16	0.49±0.14
**Ch8**	1.75±0.36	0.98±0.09	1.00±0.11	1.14±0.10	1.04±0.17	1.14±0.17	0.49±0.14
**Ch9**	1.77±0.36	0.97±0.09	0.99±0.12	1.12±0.11	1.02±0.16	1.12±0.16	0.49±0.14
**Ch10**	1.73±0.34	0.98±0.09	1.01±0.11	1.12±0.10	1.04±0.15	1.12±0.15	0.49±0.14
**Ch11**	1.61±0.46	0.99±0.08	1.00±0.10	1.11±0.11	1.02±0.16	1.16±0.16	0.53±0.23
**Ch12**	1.66±0.29	0.99±0.07	1.01±0.11	1.15±0.15	1.22±0.36	1.19±0.20	0.48±0.11
**Ch13**	1.55±0.39	1.04±0.10	1.05±0.13	1.11±0.10	1.28±0.54	1.20±0.13	0.56±0.23
**Ch14**	1.91±0.58	1.01±0.08	1.03±0.20	1.11±0.11	1.04±0.16	1.12±0.15	0.48±0.14
**Ch15**	1.74±0.35	0.99±0.09	1.05±0.10	1.11±0.11	1.04±0.17	1.12±0.17	0.48±0.14
**Ch16**	1.72±0.34	0.99±0.08	1.00±0.12	1.11±0.11	1.03±0.16	1.12±0.16	0.48±0.14

**Fig 3:**
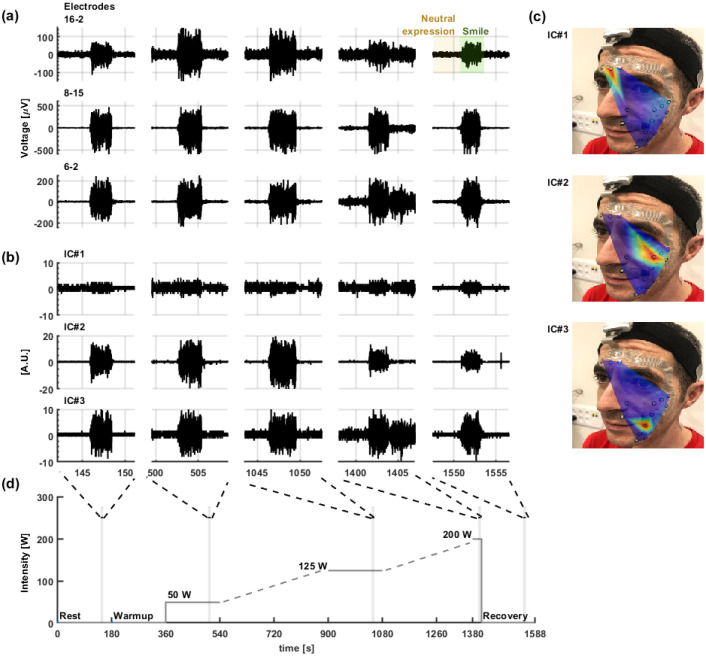
Examples of post-processing of data recorded of voluntary smiles during the exercise test. Differential sEMG (filtered) data from electrode pairs **(A)**; 3 fastICA that was applied upon the signals recorded from all 16 channels **(B)**; Corresponding heatmaps that depict the activated muscles resulted from the ICA analysis **(C)**; The cycling protocol with marks of the instructed voluntary smiles (shadowed green) **(D)**.

To further substantiate the stability of the signals in Fig 3 we show an example of post-processed data for high-resolution exploration. Specifically, we examined temporal and spatial differences in facial expressions (neutral expressions and smiles) during exercise at five different intensities: Rest, light (50 W), moderate (125 W), maximal intensity (225 w), and recovery (Fig 3d). Fig 3a shows differential signals of both a neutral expression and a voluntary smile at different stages. Differential signals between electrodes positioned at close proximity to each other can differentiate a smile from a neutral expression in all intensities (for example, Ch#6–Ch#2). Differential data help achieve amplification: One such example is the differential signal between the corrugator supercilii and zygomaticus muscle regions (Ch#15 and Ch#8, respectively) appear to be optimal for smile detection. Further insight can be gained by applying the fast independent component analysis (fastICA) algorithm. Fig 3b and 3c show an example of EMG sources extracted by the fastICA algorithm, at five different intensities. The corresponding heat-maps (Fig 3c), are consistent with specific muscles: corrugator supercilii (frowning expression, IC#1); zygomaticus (IC#2); and depressor anguli oris (IC#3). ICA data help address the cross-talk challenge, in particular for strong muscle activation, as in these physical exercising experiments. The ICA maps also clearly reveal specific muscle units, consistent with those identified under static conditions [29]. Here we demonstrate, for the first time, such mapping during physical exercise, further establishing the stability and reliability of the recorded sEMG data.

**Fig 4:**
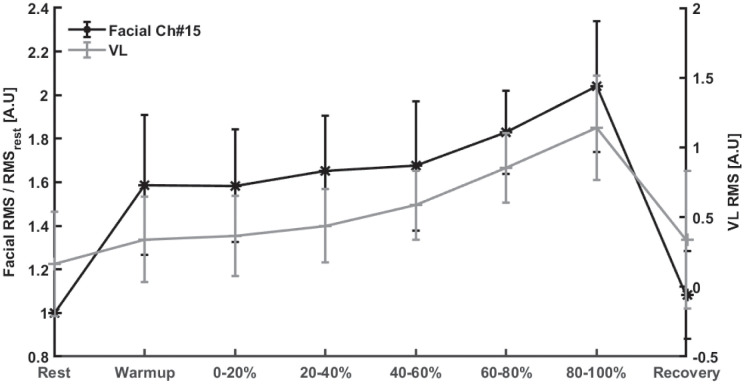
Average±SD of normalized RMS values of sEMG data from the frontalis muscle during voluntary smiles along the exercise test (black line) and the average RMS values of the VL muscle (grey line) (N = 6).

Finally, the correlation between the RMS of muscle activation during smiling and the RMS of the EMG of the VL muscle was calculated. Fig 4 depicts the dynamics of both Ch#15 (frontalis muscle) and the VL, along the exercise test (from rest, through different intensities and recovery). It is readily apparent that the frontalis muscle activation follows the pattern of the VL muscle, one of the most dominant muscles that are activated during cycling, with a positive correlation of 0.84 between the two (p < 0.05). The correlation between the VL muscle and the remaining facial muscles is provided in Table 3.

**Table 3:** Correlation coefficient table between normalized RMS values of sEMG data of each channel and RMS of VL muscle.

	Correlation Coefficient
Ch1	0.81*
Ch2	0.95*
Ch3	0.88*
Ch4	0.42
Ch5	0.67
Ch6	0.86*
Ch7	0.54
Ch8	-0.02
Ch9	0.55
Ch10	0.57
Ch11	0.69
Ch12	0.93*
Ch13	0.86*
Ch14	0.82*
Ch15	0.84*
Ch16	0.80*

*p_value_ < 0.05

## Discussion and conclusions

Our main aim in the current study was to assess the ability to faithfully record facial electromyography during exercise using soft and wearable electrode arrays. Indeed, stable recordings of facial muscle activity were achieved during a graded cycling exercise test until exhaustion. The results of this study substantiate the feasibility of recording high-resolution facial sEMG signals from both neutral expressions and voluntary smiles while exercising at different intensities in controlled settings without mechanical noise or cross talk. We also presented several examples of different signal analysis approaches for further exploration of facial expressions during exercise (from rest to exhaustion).

The soft nature of the soft multi-electrode array and the overall user convenience of the system allow an ecological assessment of facial muscle activity during challenging conditions, such as intense exercise. To demonstrate the robustness and the stability of the sensor during exercise (despite high mechanical impact during graded cycling test until exhaustion), filtered signals and the corresponding PSD were shown for different exercise intensities, even with intense head movements (Fig 2). The PSD was low at rest, as expected, while towards maximal intensity, higher PSD was observed, as participants presented pronounced facial expressions at maximal intensity, similar to the violent effort or the advanced fatigue expressions that were described by McKenzie [32]. Overall, the observed signal and PSD were similar to previously described data recorded during static settings [33].

The facial sEMG data we collected were analyzed primarily with conventional tools [18], i.e. differential (filtered) signals (Fig 3a). The segments of differential pairs showed signals with low RMS at neutral expression (“silent” face), and different patterns of muscle activation during instructed smiles, depending on exercise intensity. A differential signal of electrodes #16-#2 examines the crosstalk between the lower facial muscles to the corrugator (supercilii and procerus) muscles. Differential signals #6-#2 and #8-#15 demonstrate the increased involvement of the depressor and zygomaticus muscles, respectively, during a graded exercise test. We chose to show those muscles owing to their important role, as was previously reported. For example, Morree and Marcora [6, 7] showed the involvement of corrugator muscles during physical tasks. It was also previously shown that muscular activity of the masseter muscles (jaw clenching) and the epicranius and zygomatic muscles (the masseter muscle) were positively correlated with the degree of exertion and other fatigue-related measures during incremental workload cycling [8] and a hand weightlifting exercise [10].

Exploring these phenomena in detail can be achieved using fastICA analysis. Indeed, we showed that the fast ICA algorithm successfully separated the recorded data from the sensor’s 16 channels into independent activation sources. Three independent muscle sources are shown in Fig 3c. IC#1, IC#2 and IC#3 represent the corrugator, zygomaticus, and depressor muscle components, respectively. Our data show that the EMG signal of the frontalis area (frowning) was relatively low during rest and light intensity while increasing with exercise intensity, reaching maximal values at maximal power output. With recovery after ceasing the test, the signal decreased. Our results are in accordance with data presented a decade ago by Morree and Marcora, who were the first, to the best of our knowledge, to study the correlation between exercise exertion and facial EMG [6]. It has been concluded that frowning muscle activity reflects effort during physical tasks. Moreover, it was shown that frowning activity has the potential to differentiate exercise intensities during constant workload cycling. The zygomaticus involvement during graded exercise was also reported previously, as it was found to be positively correlated with the degree of exertion and other fatigue-related measures during incremental workload cycling [8] and a hand weightlifting exercise [10].

The RMS values of the smile events extracted from the 16-channel soft fEMG array were found to be highly correlated to VL RMS values, throughout the exercise test. RMS of the sEMG signal increased with the increased exercise intensity and decreased with recovery. It has been suggested that the increase in facial muscle activity with exercise may be a result of motor irradiation, which refers to the involuntary activation of facial muscles, which may accompany the production of voluntary activation of actual working muscles [6, 7]. The results we show here are consistent with these findings.

Despite the growing interest in monitoring facial muscle activity during exercise, there are still many challenges in recording high-quality facial muscle activity signals during exercise. It is apparent that the main challenge is the ability to record multiple facial muscles in a convenient and accurate method. This is especially important during prolonged dynamic and intense exercise, as it is difficult to run, cycle, or perform complex resistance exercises with conventional sEMG electrodes attached to one’s face. This study’s significance lies in the usage of a novel wearable multi-electrode array, allowing accurate and continuous facial electromyography during dynamic exercise (Table 4). Moreover, this sensor is unique as it allows simultaneous recording of multiple facial muscles, in high resolution, for a full facial expression mapping in the context of physical exercise. The new wearable sensor enables the design of studies with high complexity and with high relevancy for athletes, as it can be easily applied during exercise with minimal interference to the athlete.

**Table 4:** Comparison between various facial expression studies during physical tasks using facial electromyography.

Source	Exercise type	Electrodes	Monitored muscles
Morree & Marcora [6]	Leg extensions	1 Ag/AgCl electrodes	Corrugator supercilii
Morree & Marcora [7]	Cycling test with constant-workload	1 Ag/AgCl electrodes	Corrugator supercilii
Huang et al. [8]	Incremental cycling test to exhaustion	2 Ag/AgCl electrodes	Corrugator supercilii and masseter muscle
Uchida et al. [10]	Weightlifting (Arm curl lifting)	2 Ag/AgCl electrodes	Zygomatic major and epicranius muscles
This study	Incremental cycling test to exhaustion	16 Dry electrodes	Multiple facial muscles (see Table 1)

There are some limitations in this study. First, the same electrode design was used for all participants, regardless of anatomical differences (head and face) between participants. Variability in head and face anthropometrics should be considered when designing electrode arrays for different applications. Nevertheless, our results showed a high correlation between facial sEMG and the reference VL electrode. Second, as for a feasibility study, we had a small sample size from both genders, limiting the generalizability of the findings. Furthermore, because of fitness differences and possible differences in how exercise affects facial expressions, further research is needed to investigate the relationship between facial muscle activity and exercise, while focusing on objective and subjective reports. Those studies are currently underway.

To conclude, the wearable facial technology is easy to use and allows the recording of high-resolution facial sEMG while performing physical tasks. User convenience, during cycling exercise, together with good signal stability, despite high mechanical noise during exercise, are critical parameters in determining the ability to study facial expression during exercise in an ecological environment. The study’s findings may have implications in sports medicine and exercise physiology, specifically in monitoring exercise intensity and fatigue.

## References

[1] Sun W, Guo Z, Yang Z, Wu Y, Lan W, Liao Y, et al. A Review of Recent Advances in Vital Signals Monitoring of Sports and Health via Flexible Wearable Sensors. Sensors. 2022;22(20):7784. doi: 10.3390/s22207784 36298135 PMC9607392

[2] Carrier B, Barrios B, Jolley BD, Navalta JW. Validity and Reliability of Physiological Data in Applied Settings Measured by Wearable Technology: A Rapid Systematic Review. Technologies. 2020;8(4):70. doi: 10.3390/technologies8040070

[3] Adão Martins NR, Annaheim S, Spengler CM, Rossi RM. Fatigue Monitoring Through Wearables: A State-of-the-Art Review. Frontiers in Physiology. 2021;12(December). doi: 10.3389/fphys.2021.790292 PMC8715033 34975541

[4] Seshadri DR, Li RT, Voos JE, Rowbottom JR, Alfes CM, Zorman CA, et al. Wearable sensors for monitoring the internal and external workload of the athlete. npj Digital Medicine. 2019;2(1). doi: 10.1038/s41746-019-0149-2 PMC6662809 31372506

[5] Veldhuizen IJT, Gaillard AWK, De Vries J. The influence of mental fatigue on facial EMG activity during a simulated workday. Biological Psychology. 2003;63(1):59–78. doi: 10.1016/S0301-0511(03)00025-5 12706964

[6] de Morree HM, Marcora SM. The face of effort: Frowning muscle activity reflects effort during a physical task. Biological Psychology. 2010;85(3):377–382. doi: 10.1016/j.biopsycho.2010.08.009 20832447

[7] De Morree HM, Marcora SM. Frowning muscle activity and perception of effort during constant-workload cycling. European Journal of Applied Physiology. 2012;112(5):1967–1972. doi: 10.1007/s00421-011-2138-2 21879350

[8] Huang DH, Chou SW, Chen YL, Chiou WK. Frowning and jaw clenching muscle activity reflects the perception of effort during incremental workload cycling. Journal of Sports Science and Medicine. 2014;13(4):921–928. 25435786 PMC4234963

[9] Blanchfield AW, Hardy J, De Morree HM, Staiano W, Marcora SM. Talking yourself out of exhaustion: The effects of self-talk on endurance performance. Medicine and Science in Sports and Exercise. 2014;46(5):998–1007. doi: 10.1249/MSS.0000000000000184 24121242

[10] Uchida MC, Carvalho R, Tessutti VD, Bacurau RFP, Coelho-Júnior HJ, Capelo LP, et al. Identification of muscle fatigue by tracking facial expressions. PLoS ONE. 2018;13(12):1–11. doi: 10.1371/journal.pone.0208834 30562370 PMC6298643

[11] Khanal SR, Sampaio J, Barroso J, Filipe V. Individual’s Neutral Emotional Expression Tracking for Physical Exercise Monitoring; 2020. p. 145–155. Available from: http://link.springer.com/10.1007/978-3-030-60117-1_11.

[12] Huang CN, Chen CH, Chung HY. The review of applications and measurements in facial electromyography. Journal of Medical and Biological Engineering. 2005;25(1):15–20.

[13] Kreis C, Aguirre A, Cifuentes CA, Munera M, Jiménez MF, Schneider S. Predicting Perceived Exhaustion in Rehabilitation Exercises Using Facial Action Units. Sensors. 2022;22(17):6524. doi: 10.3390/s22176524 36080983 PMC9459962

[14] Miles KH, Clark B, Périard JD, Goecke R, Thompson KG. Facial feature tracking: a psychophysiological measure to assess exercise intensity? Journal of Sports Sciences. 2018;36(8):934–941. doi: 10.1080/02640414.2017.1346275 28665235

[15] Zhu T, Zhang C, Wu T, Ouyang Z, Li H, Na X, et al. Research on a Real-Time Driver Fatigue Detection Algorithm Based on Facial Video Sequences. Applied Sciences (Switzerland). 2022;12(4). doi: 10.3390/app12042224

[16] Gat L, Gerston A, Shikun L, Inzelberg L, Hanein Y. Similarities and disparities between visual analysis and high-resolution electromyography of facial expressions. PLOS ONE. 2022;17(2):e0262286. doi: 10.1371/journal.pone.0262286 35192638 PMC8863227

[17] Cifrek M, Medved V, Tonković S, Ostojić S. Surface EMG based muscle fatigue evaluation in biomechanics. Clinical Biomechanics. 2009;24(4):327–340. doi: 10.1016/j.clinbiomech.2009.01.010 19285766

[18] De Luca CJ. The use of surface electromyography in biomechanics. Journal of Applied Biomechanics. 1997;13(2):135–163. doi: 10.1123/jab.13.2.135

[19] Ament W, Bonga GJJ, Hof AL, Verkerke GJ. EMG median power frequency in an exhausting exercise. Journal of Electromyography and Kinesiology. 1993;3(4):214–220. doi: 10.1016/1050-6411(93)90010-T 20870536

[20] Bonato P, Roy SH, Knaflitz M, De Luca CJ. Time frequency parameters of the surface myoelectric signal for assessing muscle fatigue during cyclic dynamic contractions. IEEE Transactions on Biomedical Engineering. 2001;48(7):745–753. doi: 10.1109/10.930899 11442286

[21] Merletti R, Lo Conte LR. Surface EMG signal processing during isometric contractions. Journal of Electromyography and Kinesiology. 1997;7(4):241–250. doi: 10.1016/S1050-6411(97)00010-2 11369267

[22] Wan JJ, Qin Z, Wang PY, Sun Y, Liu X. Muscle fatigue: General understanding and treatment. Experimental and Molecular Medicine. 2017;49(10):384–11. doi: 10.1038/emm.2017.194 28983090 PMC5668469

[23] Tenan MS, McMurray RG, Troy Blackburn B, McGrath M, Leppert K. The relationship between blood potassium, blood lactate, and electromyography signals related to fatigue in a progressive cycling exercise test. Journal of Electromyography and Kinesiology. 2011;21(1):25–32. doi: 10.1016/j.jelekin.2010.09.002 20934353

[24] González-Izal M, Malanda A, Gorostiaga E, Izquierdo M. Electromyographic models to assess muscle fatigue. Journal of Electromyography and Kinesiology. 2012;22(4):501–512. doi: 10.1016/j.jelekin.2012.02.019 22440555

[25] Zhao Y, Wang J, Zhang Y, Liu H, Chen Z, Lu Y, et al. Flexible and Wearable EMG and PSD Sensors Enabled Locomotion Mode Recognition for IoHT-Based In-Home Rehabilitation. IEEE Sensors Journal. 2021;21(23):26311–26319. doi: 10.1109/JSEN.2021.3058429

[26] Fang C, Wang Y, Gao S. Flexible and Wearable GRF and EMG Sensors Enabled Locomotion Mode Recognition for IoHT Based In-home Flexible and Wearable GRF and EMG Sensors Enabled Locomotion Mode Recognition for IoHT Based In-home Rehabilitation. TechRxiv Preprint. 2020. 10.36227/techrxiv.11987994.v1

[27] Bareket L, Inzelberg L, Rand D, David-Pur M, Rabinovich D, Brandes B, et al. Temporary-tattoo for long-term high fidelity biopotential recordings. Scientific Reports. 2016;6(December 2015):1–8. doi: 10.1038/srep25727 27169387 PMC4864418

[28] Inzelberg L, David Pur M, Steinberg S, Rand D, Farah M, Hanein Y. Wireless electronic-tattoo for long-term high fidelity facial muscle recordings. In: George T, Dutta AK, Islam MS, editors. Micro- and Nanotechnology Sensors, Systems, and Applications IX. vol. 10194; 2017. p. 101940U. Available from: http://proceedings.spiedigitallibrary.org/proceeding.aspx?doi=10.1117/12.2263522.

[29] Inzelberg L, David-Pur M, Gur E, Hanein Y. Multi-channel electromyography-based mapping of spontaneous smiles. Journal of Neural Engineering. 2020;17(2). doi: 10.1088/1741-2552/ab7c18 32271717

[30] Inzelberg L, Rand D, Steinberg S, David-Pur M, Hanein Y. A Wearable High-Resolution Facial Electromyography for Long Term Recordings in Freely Behaving Humans. Scientific Reports. 2018;8(1):1–9. doi: 10.1038/s41598-018-20567-y 29391503 PMC5794977

[31] Shustak S, Inzelberg L, Steinberg S, Rand D, David Pur M, Hillel I, et al. Home monitoring of sleep with a temporary-tattoo EEG, EOG and EMG electrode array: A feasibility study. Journal of Neural Engineering. 2019;16(2). doi: 10.1088/1741-2552/aafa05 30566912

[32] M Kenzie RT. The Facial Expression of Violent Effort, Breathlessness, and Fatigue. Journal of anatomy and physiology. 1905;40(Pt 1):51–56. 17232662 PMC1287338

[33] Boxtel AV. Facial EMG as a tool for inferring affective states. Proceedings of measuring behavior. 2010;2010(August 24-27):104–108.

